# Cloning and Expression Analysis of a *PISTILLATA* Homologous Gene from Pineapple (*Ananas comosus* L. Merr)

**DOI:** 10.3390/ijms13011039

**Published:** 2012-01-19

**Authors:** Ling-Ling Lv, Jun Duan, Jiang-Hui Xie, Yu-Ge Liu, Chang-Bin Wei, Sheng-Hui Liu, Jian-Xia Zhang, Guang-Ming Sun

**Affiliations:** 1 South Subtropical Crops Research Institute, Chinese Academy of Tropical Agricultural Science, Zhanjiang 524091, China; E-Mails: lulingling1234@21cn.com (L.-L.L.); Xiejianghui@21cn.com (J.-H.X.); liuyugehb@126.com (Y.-G.L.); zbwcb@163.com (C.-B.W.); Lizx2002@126.com (S.-H.L.); 2 South China Botanical Garden, Chinese Academy of Sciences, Guangzhou 510650, China; E-Mails: duanj@scib.ac.cn (J.D.); zhangjianxia@163.com (J.-X.Z.)

**Keywords:** pineapple, flower development, *PI*-like, cloning, expression, transformation

## Abstract

*PISTILLATA* (*PI*)*-*like genes are crucial regulators of flowering in angiosperms. A homologue of *PI*, designated as *AcPI* (Genbank accession number HQ717796), was isolated from pineapple cultivar Comte de Paris by reverse transcriptase polymerase chain reaction (RT-PCR) and rapid amplification of cDNA ends (RACE). The cDNA sequence of *AcPI* is 907 bp in length and contains an open reading frame of 594 bp, which encodes a protein of 197 amino acids. The molecular weight was 2.29 kDa and the isoelectric point was 9.28. The alignment showed that *AcPI* had a high identity with CsPIC2 (78.6%), AoPI (77.4%), OrcPI (75.7%) and HPI2 (72.4%). Quantitative real-time polymerase chain reaction (qRT-PCR) analyses in different tissues showed that the expression pattern of *AcPI* was different from the B-class genes in eudicots. *AcPI* was expressed in all the tissues investigated. The expression level was very low in fruit stems, bracts, leaves and sepals, high in petals and carpels, and moderate in apical meristems, flesh and stamens. The qRT-PCR analyses in different stages indicated that the expression of *AcPI* reached the highest level at 40 days after flower inducement, when the multiple fruit and floral organs were forming. It proved the important role of *AcPI* in floral organs and fruit development. The 35S::*AcPI* transgenic *Arabidopsis* plants flowered earlier and had more inflorescences or branches than wild type plants.

## 1. Introduction

Most angiosperm flowers, including those of pineapple, are made up of four types of organs that are arranged in concentric whorls from outside to inside: sepals, petals, stamens, and the inner carpels [1]. According to the widely accepted ABC model [2,3] of floral organ development, there are three classes of homeotic genes: A-class, B-class and C-class [4–6]. Expression of the B-class genes, such as the *Arabidopsis APETALA3* (*AP3*) and *PISTILLATA* (*PI*) as well as the *Antirrhinum DEFICIENS* (*DEF*) and *GLOBOSA* (*GLO*), two closely related clades of MADS-box genes, is required for petal and stamen initiation and development [7,8]. Other angiosperms and gymnosperm further possess a sister clade of B genes, termed Bsister genes, and expression studies revealed that these genes are predominantly expressed in carpels and ovules [9]. Mutations in these genes cause homeotic conversion of petals in the second whorl to sepals and of stamens in the third whorl to carpels [10,11].

MADS-box is a recognized type of DNA-binding protein [4] including around 60 amino acids, that are highly conserved across developmental control genes from yeast, animals and plants [12–14]. All MADS-box proteins encode transcription factors that regulate the expression of target genes by binding to specific *cis*-acting DNA sequences. The MADS-box protein plays fascinating biological roles in flower development in higher plants [15].

In contrast to the great amount of research conducted on MADS-box genes in dicots, a limited number of MADS-box genes have been studied in monocots. Moreover, the reported data of MADS-box genes in monocots are mostly concerned with economically important plants such as rice and maize [16–22]. Few data are available for other monocots [23–26]. More work to assess the degree to which the dicot ABCDE model [27] is conserved in monocot species needs to be done.

Pineapple (*Ananas comosus* L. Merr.) belongs to *Ananas*, *Bromeliaceae*, *Poales*. It is an important herbaceous fruit tree in many tropical and subtropical countries. The pineapple fruit is a multiple fruit, which includes over one hundred small flowers and each flower develops into a small fruit. There are three sepals, three petals, six stamens, one pistil and three carpels in each flower. Nutritionally, freshly harvested pineapple is an inexpensive source of vitamins A, B, and C, calcium, phosphorus and iron. The fruit is used for canning and in the preparation of juice, jam, jelly and crystallized glaze fruit. The leaves, stems and fruits of the pineapple plants contain *bromelain* which is a mixture of industrially important proteolytic enzymes. This is the reason why several research groups are developing fundamental and applied studies to create new cultivars with better agronomic characters. However, natural flowering out of season can cause serious scheduling problems for growers. Flower inducing is a good way to make pineapples flower at the same time, however, with some species it is difficult to induce flowering.

Like other monocot plants, such as lily, pineapple flowers have very similar organs of two outer whorls, generating a perianth of tepals, instead of sepals and petals. In order to elucidate the genetic mechanism of the pineapple flower development, cloning and characterization of MADS-box genes from pineapple is a fundamental work. In this study, a *PI*-like gene was cloned and its expression analyses were also studied.

## 2. Results and Discussion

### 2.1. Cloning and Sequence Analyses of *AcPI*

**Figure 1:**
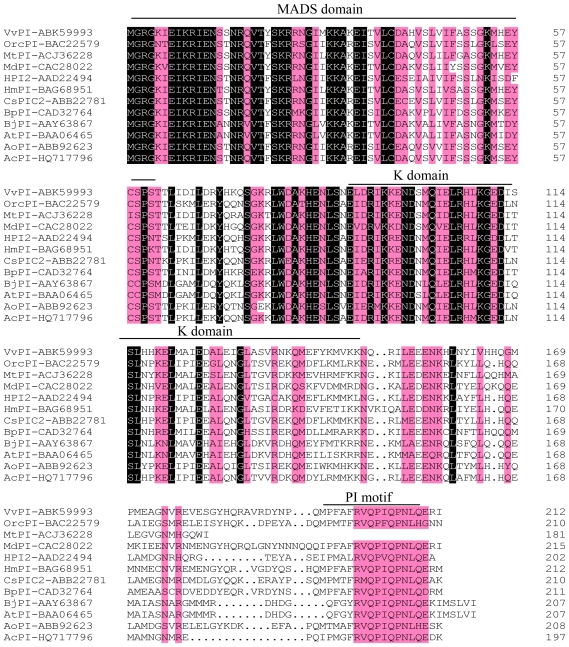
Alignment of the deduced *AcPI* (Genbank accession number HQ717796) amino acid sequence from *Ananas comosus* with other angiosperm *PISTILLATA* (*PI*)*-*like protein sequences. Identical amino acid residues in this alignment are shaded in black, and 75% or more similar amino acid residues are shaded in red.

A combined reverse transcriptase polymerase chain reaction (RT-PCR) and rapid amplification of cDNA ends (RACE) strategy was used to isolate *PI*-like gene from pineapple. The full-length gene designated *AcPI* (Genbank accession number HQ717796) was isolated. *AcPI* cDNA is 907 bp in length and contains an open reading frame of 594 bp, which encodes a protein of 197 amino acids. It also contains 101 bp 5′ and 183 bp 3′ untranslated regions and a poly (A) tail. Molecular weight was 2.29 kDa and isoelectric point was 9.28. A 650 bp fragment of *AcPI* with enzyme recognition sites and coding region was obtained and used for transformation. An alignment of the deduced amino acid sequence of *AcPI* (*Ananas comosus*), HPI2 (*Hyacinthus orientalis*, AAD22494), VvPI (*Vitis vinifera*, ABK59993), BpPI (*Betula pendula*, CAD32764), MdPI (*Malu* × *domestica*, CAC28022), HmPI (*Hydrangea macrophylla*, BAG68951), BjPI (*Brassica juncea*, AAY63867), AtPI (*Arabidopsis thaliana*, BAA06465), MtPI (*Medicago truncatula*, ACJ36228), OrcPI (*Orchis italica*, BAC22579), CsPIC2 (*Crocus sativus*, ABB22781) and AoPI (*Alpinia oblongifolia*, ABB92623) was performed using the DNAMAN 6.0 program (Figure 1).

**Figure 2:**
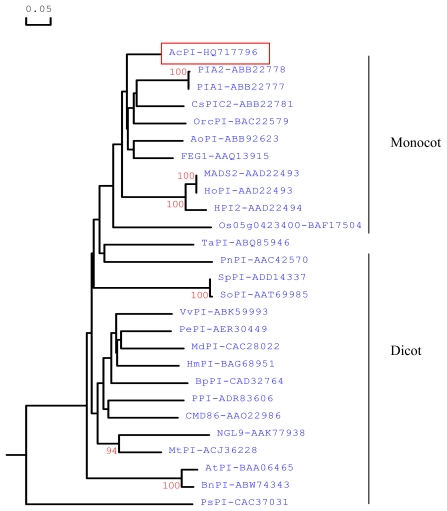
Phylogenetic analysis of the *PI* homologous genes from different plant species. Protein sequences of the entire coding region were obtained from the NCBI database. The numbers next to the nodes give bootstrap values from 1000 replicates and the branch lengths are proportional to the distance.

Phylogenetic analysis was conducted using BnPI (*Brassica napus*, ABW74343), PnPI-1 (*Papaver nudicaule,* AAC42570), CDM86 (*Chrysanthemum × morifolium*, AAO22986)*,* SoPI (*Spinacia oleracea*, AAT69985), PePI (*Passiflora edulis*, AER30449), PPI (*Capsicum annuum*, ADR83606), NGL9 (*Medicago sativa*, AAK77938), PsPI (*Pisum sativum*, CAC37031), TaPI (*Trochodendron aralioides*, ABQ85946), HoPI (*Hyacinthus orientalis*, AAD22494), Os05g0423400 (*Oryza sativa Japonica Group*, BAF17504), FEG1 (*Elaeis guineensis*, AAQ13915), PIA2 (*Crocus sativus*, ABB22778), PIA1 (*Crocus sativus*, ABB22777), MADS2 (*Hyacinthus orientalis*, AAD22493) and the same complete amino acid sequences as in Figure 1 and generated a rooted tree, by the observed divergency method with DNAMAN 6.0 program (Figure 2). The phylogenetic tree showed that *AcPI* protein (*red-boxed*) was more closely related to the other monocot *PI* proteins than to their dicot counterparts. *AcPI* had a high identity with CsPIC2 (78.6%), AoPI (77.4%), OrcPI (75.7%) and HPI2 (72.4%).

### 2.2. The qRT-PCR Analyses of *AcPI*

**Figure 3:**
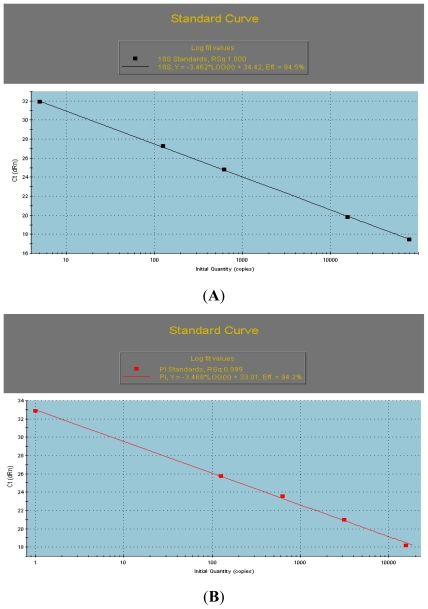

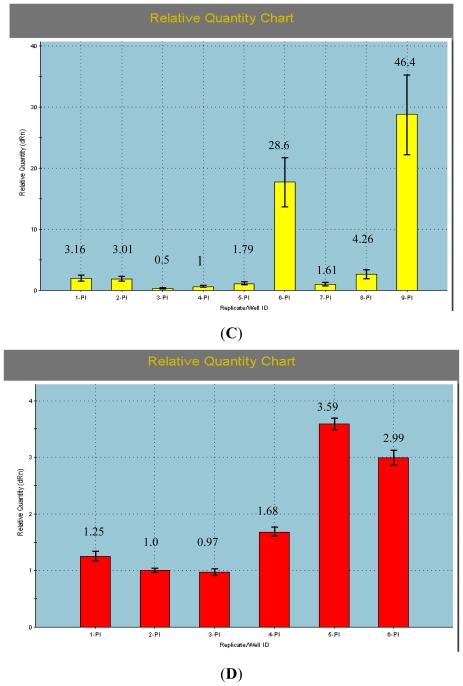
(**A**) The standard curve of *Ac 18S rRNA*. (**B**) The standard curve of *AcPI*. (**C**) Quantification of expression levels of the *AcPI* gene in different tissues as determined by qRT-PCR analyses. The housekeeping gene *Ac 18S rRNA* was used to normalize the amount of cDNAs added to the reaction. The relative quantification values of different tissues were shown above each vertical bar. 1-PI, apical meristems; 2-PI, flesh; 3-PI, fruit stems; 4-PI, bracts; 5-PI, leaves; 6-PI, petals; 7-PI, sepals; 8-PI, stamens; 9-PI, carpels. (**D**) The quantification of expression levels of the *AcPI* gene in different stages as determined by qRT-PCR analyses. The housekeeping gene *Ac 18S rRNA* was used to normalize the amount of cDNAs added to the reaction. The relative quantification values of different stages are shown above each vertical bar. 1-PI to 6-PI denote the expression levels in apical meristems at 1d before flower inducing, 10 days, 20 days, 30 days, 40 days and 50 days after flower inducing respectively.

To determine the detail expression patterns of the *AcPI* in different tissues and in different stages, the mRNA levels were examined by qRT-PCR. Relative quantification was performed using the comparative cycle threshold (Ct) method, in which simplified arithmetic formulas (2-ΔΔCt) are used to obtain the same result as that yielded by the relative standard curve method, when the target gene and the reference control gene have approximately equal amplification efficiency (94.2% and 94.5% respectively, Figure 3A,B). The expression number in one tissue or stage (*AcPI* in bract and on 10 days after inducing) was set to a value of 1 and subsequently, expression levels were relative to this number. The qRT-PCR analyses showed that the *AcPI* was weakly expressed in fruit stems, bracts, leaves and sepals, strongly in petals and carpels, and moderately in apical meristems, flesh, and stamens (Figure 3C).

The expression levels of *AcPI* in different stages (Figure 3D) decreased a little at 10 days and 20 days after flower inducing with ethephon. It increased at 30 days after flower inducing and reached the highest level at 40 days after flower inducing when the multiple fruit and floral organs were forming.

### 2.3. Ectopic Expression of *AcPI* in Arabidopsis

**Figure 4:**
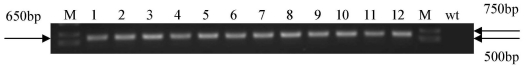
1 to 12, PCR analysis of 35S::*AcPI* transgenic *Arabidopsis* plants using primers PImq-F and PImq-R. A 650 bp DNA fragment was amplified. M, Marker DL2000. wt, PCR analysis of wild type plant using the same primers.

**Figure 5:**
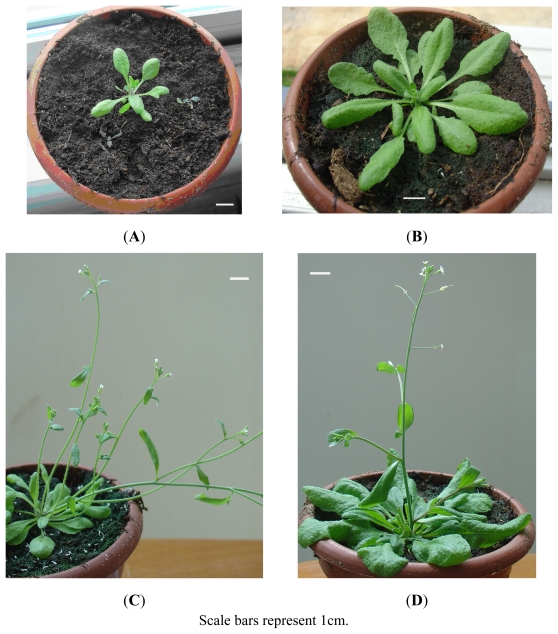
(**A**) The flower buds formation of the 35S::*AcPI* transgenic *Arabidopsis* plants. (**B**) The flower buds formation of the wild type plants. (**C**) The 35S::*AcPI* transgenic *Arabidopsis* plant with six inflorescences. (**D**) The wild type plant with one inflorescence.

To investigate the function of *AcPI*, ectopic expression of *AcPI* in transgenic *Arabidopsis* plants was analyzed. Twelve independent PCR-positive 35S::*AcPI* transgenic T1 plants were obtained (Figure 4). All transgenic plants were phenotypically distinguishable from wild type plants. Firstly, these 35S::*AcPI* transgenic plants flowered significantly earlier than wild type plants by producing only 8 to 10 small rosette leaves (Figure 5A). When the wild type plants flowered, they had over sixteen big rosette leaves (Figure 5B). Secondly, each 35S::*AcPI* transgenic plant had 3 to 6 inflorescences or branches (Figure 5C), while the wild type plant had one inflorescence or branch (Figure 5D). No obvious alteration of floral organs was observed in transgenic plants.

### 2.4. Discussion

Multiple sequence alignment demonstrated that AcPI protein possessed a typical MIKC-type domain structure: the highly conserved MADS (M) domain which is the major determinant of DNA-binding and performs dimerization and accessory factor binding functions [13], the relatively weakly conserved intervening (I) domain which constitutes a molecular determinant for the selective formation of DNA-binding dimmers [28], the less conserved keratin-like (K) domain which is proposed to allow for the formation of an amphipathic helix involved in protein dimerization [13,29], and the divergent *C*-terminal (C) region which contains a highly conserved *PI*-motif (FxFRVQPxQPNLQE) [30]. All the conserved regions are related to the function of *PI*-like proteins [31].

In higher eudicotyledonous angiosperms, the floral organs include sepals, petals, stamens, ovules and carpels. According to the ABCDE model, the identity of these organs is specified by floral homeotic genes of class A, A + B + E, B + C + E, D + E and C + E respectively [27,32]. To explain the *Liliaceae* flower morphology, van Tunen *et al.* [23] reported that class B genes were not only expressed in petals and stamens, but also in sepals. Thus the organs of both sepals and petals express class A, plus class B genes and therefore, get the same petaloid identity. Kanno A *et al.* [33] reported that two *DEF*-like and one *GLO*-like genes were expressed in whorls 1, 2 and 3. In addition, *TGGLO* was also weakly expressed in carpels, leaves, stems and bracts. Expression of B-class genes in *Arabidopsis* and *Antirrhinum* was known to be flower-specific [8, 34–36].

In this paper, the expression profile of *AcPI* is different from B-box genes of eudicot. *AcPI* was expressed not only in whorls 2 and 3, but also in whorl 1, thus corroborating the modified ABC model [23]. Moreover, *AcPI* was expressed in whorl 4. *AcPI* was also expressed in other organs or tissues such as leaves, fruit stems, flesh, apical meristems and bracts. These data indicated a possible function of *AcPI* during flower development as well as in leaves and fruit development. Busi M *et al.* [37] reported that B-box genes were involved in other processes than flower development such as the establishment of developing embryos, seed coat and ultimately in root and fruit development. Kim *et al.* [38] also found *AP3* and *PI* transcripts were detected in petals, stamens and carpels in basal angiosperms such as *Amborella* and *Nuphar*. The expression of *AcPI* reached the highest level at 40 days after flower inducement, when the multiple fruit and floral organs were forming. The results showed the important role of *AcPI* in fruit and floral organs development and indicated that ethephon may stimulate the expression of *AcPI*. Ethephon are used widely to induce flowering in pineapples, possibly because when ethephon reaches the shoot apex, it can accelerate the expression of flower-related genes [39]. The effect of ethephon on inducing flowering was affected by temperature, relative humidity, solution pH, and the surface on which solution droplets were placed [39]. The ectopic expression of *AcPI* proved that *AcPI* could accelerate flowering and inflorescences or branches forming in *Arabidopsis.*

## 3. Materials and Methods

### 3.1. Plant Materials and Treatments

Plants of pineapple cultivar Comte de Paris were planted in a vinyl house at natural temperature and light. The apical meristems of young panicle (4–5 cm in height) were cut and collected for cloning cDNA. When the young panicle was 4–5 cm in height, the apical meristems, flesh, fruit stems, bracts, leaves, petals, sepals, stamens and carpels were used for qRT-PCR analyses. The apical meristems at 1 days before flower inducing and 10 days, 20 days, 30 days, 40 days, 50 days after flower inducing with 40% ethephon (200 mg/mL, 30 mL each plant) were also used for qRT-PCR analyses. All the materials were frozen in liquid nitrogen immediately after collection and stored at −80 °C until use.

### 3.2. Cloning of *AcPI* cDNAs

Total RNA was extracted from apical meristems using Column Plant RNAout 2.0 kit (TIANDZ, Inc, China) following the manufacturer’s instructions. First-strand cDNA was synthesized with M-MLV-Reverse Trancriptase from TAKARA according to the manufacturer’s instructions.

To clone the conserved region of *AcPI* cDNA, a pair of primers PI-partial-F (5′ CCAGGTC TCCVTCGTCAT 3′) and PI-partial-R (5′ ACACCAGTNAGACCATTC 3′) was designed according to the conserved regions of *PI* homologous genes from other plants using the Primer Premier 5 software. The PCR amplification was performed with 1 cycle at 94 °C for 3 min; 32 cycles at 94 °C for 0.5 min, 55 °C for 1 min, and 72 °C for 1 min; 1 cycle at 72 °C for 10min. PCR products were isolated and cloned into pMD18-T Vector (TAKARA, Dalian, China) to sequence. The cloned sequence was used to design gene-specific primers to amplify the 5′ and 3′ end of cDNA using the RACE cDNA Amplification Kit (Clontech, Japan). The gene-specific primers were 5′PI-OUT (5′ CCA TTC TGG AGA GCC TCT TCT ATC GG 3′) and 5′PI-IN (5′ GCT CGA TCT GCA TGT TGT CGT TCT CT 3′) for 5′ RACE, 3′PI-OUT (5′ CGG GAA GAT GTC CGA GTA CTG TAG CC 3′) and 3′PI-IN (5′ AAC GAC AAC ATG CAG ATC GAG CTC AG 3′) for 3′ RACE. The first round PCR and the nested amplification were carried out according to the instruction of RACE cDNA Amplification Kit. The PCR products were cloned into pMD18-T vector and sequenced.

The full length cDNA of *AcPI* was obtained by PCR using the forward primer PIQC-F (5′ AAG CAG TGG TAT CAA CGC AGA GTA 3′) and the reverse primer PIQC-R (5′ ATA GCA GAC AAA GTC GAT GGC AGA 3′). Another pair of primers PImq-F (5′ TGC TCTAGA ATG GGG CGG GGG AAG ATC GAG AT 3′) and PImq-R (5′ CG GGATCC ATA GCA GAC AAA GTC GAT GGC AGA 3′) contained the generated *Xba*I and *Bam*HI recognition sites respectively to facilitate the transformation of *AcPI* into *Arabidopsis*. The cycling condition:1 cycle at 94 °C for 3 min; 35 cycles at 94 °C for 0.5 min, 50 °C for 1.5 min and 72 °C for 1 min; a final extension at 72 °C for 10 min. The PCR products were cloned into pMD18-T vector and sequenced.

### 3.3. Sequence and Phylogenetic Analyses

Sequence chromatograms were examined and edited using Chromas Version 2.23. Related sequences were found using BLAST [40]. Amino acid alignments with homologous sequences from a range of species were manually edited with the DNAMAN 6.0 program. For determination of amino acid identities, sequences taken from the alignment were pairwise-compared using DNAMAN 6.0. A phylogenetic tree, based on the amino acid sequences, was constructed using DNAMAN 6.0. Gaps appearing in one sequence only, were treated as non-constant characters. The molecular weight and isoelectric point of the gene were analyzed on-line with ExPASy [41].

### 3.4. qRT-PCR Analyses of the *AcPI* Gene

To study transcription of the *AcPI* gene by the qRT-PCR, total RNA of the apical meristems in different stages of flesh, fruit stems, bracts, leaves, petals, sepals, stamens and carpels was extracted with Column Plant RNAout 2.0 kit (TIANDZ, Inc, China) following the manufacturer’s instructions. First-strand cDNA was synthesized with PrimeScript^®^ RT Master Mix (Perfect Real Time, TAKARA, Dalian, China) according to the user manual. These cDNAs were used as templates for qRT-PCR. Each tissue was applied to three replications for gene expression.

To demonstrate that amplification efficiencies for both the *AcPI* gene and housekeeping gene *Ac 18S rRNA* were very similar, standard curves were carried out by PCR. The curves were generated for genes *AcPI* and *Ac 18S rRNA* using a 5× serial dilution curve, with final quantities of 100, 20, 4, 0.8, 0.16 and 0.032 ng of cDNA.

qRT-PCR was carried out using SYBR^®^ Premix Ex Taq™ kit (TAKARA, Dalian, China) and the PCR amplification was quantified according to the manufacturer’s protocol. Real time PCR reactions were performed in 25 μL mixtures. The mixture for one reaction contained 12.5 μL 1× SYBR Green PCR Master Mix with 0.5 μL ROXII as a reference dye for real time PCR, 1 μL 10 μM of forward primer, 1 μL 10μM reverse primer and 100 ng of cDNA. No template controls were run to determine contamination and level of primer dimmer formation. To make it possible to compare gene expression levels in the different plant tissues, they were normalized to the expression of *Ac 18S rRNA* in each tissue. Relative expression of *AcPI* in different plant tissues was obtained by dividing the average number of copies by the copy number of *Ac 18S rRNA* for the same tissues.

qRT-PCR reactions were run on a Stratagene Mx3005P detection system (Stratagene 3005P, USA) using the following universal cycling conditions for all amplifications: 1 cycle of 30 s at 95 °C (DNA polymerase activation); 40 cycles of 5 s at 95 °C and 1 min at 55 °C. At the end, a dissociation stage was added: 30 s at 95 °C, 1min at 55 °C and 30 s at 95 °C. Ct values were determined after automatic adjustment of the baseline and manual adjustment of the fluorescence threshold.

The primers used in this qRT-PCR were listed below: PIdl-up (5′ GCA CCA CCA AGA GAT GGC GAT G 3′), PIdl-dn (5′ TAG CAG ACA AAG TCG ATG GCA GAG A 3′). *Ac 18S rRNA* was used as the housekeeping gene. *Ac 18S rRNA*-up (5′ ATG GTG GTG ACG GGT GAC 3′), *Ac 18S rRNA*-dn (5′ AGA CAC TAA AGC GCC CGG TA 3′).

### 3.5. Arabidopsis Transformation and PCR Analysis of Transgenic Plants

*AcPI* was excised from the pMD18-T vector using *Xba*I and *Bam*HI restriction enzymes and inserted into the vector pBI121 under the control of cauliflower mosaic virus 35S promoter. After confirmation of the sequence, the plant expression vector was transformed into *Agrobacterium tumefaciens* strain GV3101 via the freeze-thaw method [42]. Then the 35S::*AcPI* was transformed into *Arabidopsis thaliana* ecotype Columbia plants using a floral dip method [43].

Transformants which had survived in the 1/2 times MS medium containing kanamycin (50 mg/L) were further verified by PCR analysis. For PCR analysis, the Column Plant DNAout kit (TIANDZ, Inc, China) was used to isolate DNA from fresh leaves (100 mg) of T1 transgenic plants and non-transgenic plants. The primers were PImq-F and PImq-R. The cycling condition was the same as that used in amplification of cDNA with enzyme recognition sites.

## 4. Conclusions

An *AcPI* was cloned from pineapple. The cDNA sequence of *AcPI* is 907 bp in length and contains an open reading frame of 594 bp, which encodes a protein of 197 amino acids. The qRT-PCR analyses in different tissues showed that the expression pattern of *AcPI* was different from the B-class genes in eudicots. *AcPI* was expressed in all the tissues investigated. The expression level was very low in fruit stems, bracts, leaves and sepals, high in petals and carpels, and moderate in apical meristems, flesh and stamens. The qRT-PCR analyses in different stages indicated that the expression of *AcPI* reached the highest level at 40 days after flower inducement, when the multiple fruit and floral organs were forming. The 35S::*AcPI* transgenic *Arabidopsis* plants flowered earlier and had more inflorescences or branches than the wild type plants.
